# The genome sequence of the Squinting Bush Brown, *Bicyclus anynana* (Butler, 1879)

**DOI:** 10.12688/wellcomeopenres.19432.1

**Published:** 2023-06-27

**Authors:** Ilik J. Saccheri, Wellcome Sanger Institute Tree of Life programme, Wellcome Sanger Institute Scientific Operations: DNA Pipelines collective, Tree of Life Core Informatics collective, Darwin Tree of Life Consortium

**Affiliations:** 1 Department of Evolution, Ecology and Behaviour, University of Liverpool, Liverpool, England, UK

**Keywords:** Bicyclus anynana, Squinting Bush Brown, genome sequence, chromosomal, Lepidoptera

## Abstract

We present a genome assembly from an individual female *Bicyclus anynana *(the Squinting Bush Brown; Arthropoda; Insecta; Lepidoptera; Nymphalidae). The genome sequence is 457.2 megabases in span. Most of the assembly is scaffolded into 28 chromosomal pseudomolecules, including the Z sex chromosome. The mitochondrial genome has also been assembled and is 16.1 kilobases in length.

## Species taxonomy

Eukaryota; Metazoa; Ecdysozoa; Arthropoda; Hexapoda; Insecta; Pterygota; Neoptera; Endopterygota; Lepidoptera; Glossata; Ditrysia; Papilionoidea; Nymphalidae; Satyrinae; Satyrini; Mycalesina; *Bicyclus*; *Bicyclus anynana* (Butler, 1879) (NCBI:txid110368).

## Background

**Figure 1.**
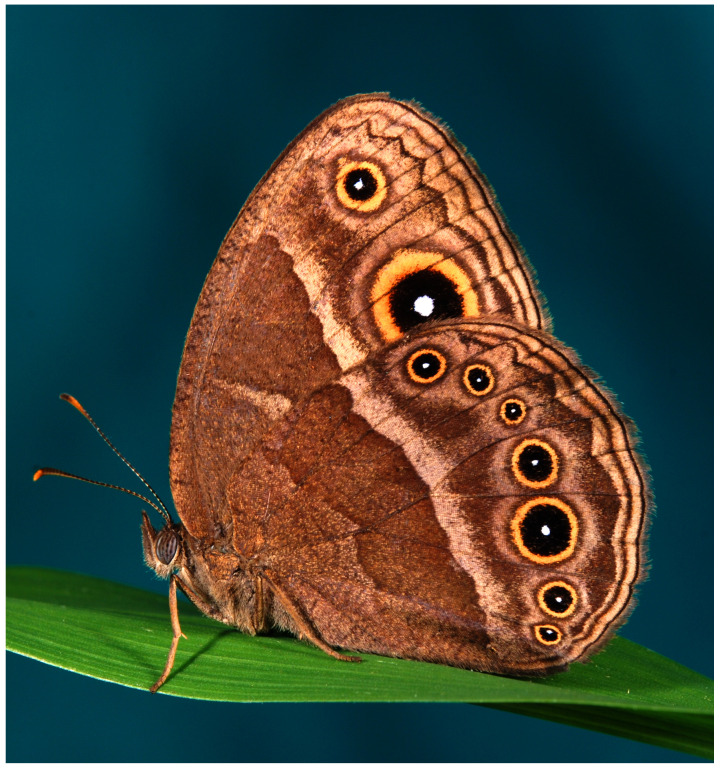
*Bicyclus anynana* female – wet season phenotype. (Photograph by William Piel, source: https://lepdata.org/monteiro/PNAS%20covers%20shots.html)

*Bicyclus anynana*, commonly known as the Squinting Bush Brown, is a widely distributed, medium-sized, Afrotropical satyrid butterfly, inhabiting grasslands and forest edges. Its seasonally plastic phenotype and modular pattern of wing eye spots ( Figure 1), combined with ease of rearing in captivity, have made it a model system for evo-devo and life-history research ( Brakefield *et al.*, 2009). In nature, the primary larval host plant is *Oplismenus compositus,* and the adults feed on rotting fruit; the laboratory stocks have been domesticated onto maize and wheatgrass.

The standard karyotype consists of WZ females and ZZ males ( *n* = 28), but the W chromosome is exceptionally small and remains to be assembled ( Beldade *et al.*, 2009; Van’t Hof *et al.*, 2008). The present genome is the third independent assembly ( Murugesan *et al.*, 2022; Nowell *et al.*, 2017), which will support ongoing research on a wide range of fundamental questions. These include the genetics and development of wing colour pattern ( Prakash *et al.*, 2022), regulation of phenotypic plasticity ( Tian & Monteiro, 2022), responses to climate change ( Oostra *et al.*, 2018), genetics of inbreeding depression ( Saccheri *et al.*, 2020), and sex-determination mechanisms.

## Genome sequence report

The genome was sequenced from one female *Bicyclus anynana* from a collection at the University of Liverpool. A total of 20-fold coverage in Pacific Biosciences single-molecule HiFi long reads was generated. Primary assembly contigs were scaffolded with chromosome conformation Hi-C data. Manual assembly curation corrected 117 missing joins or mis-joins and removed 6 haplotypic duplications, reducing the scaffold number by 32.52%. 

**Figure 2.**
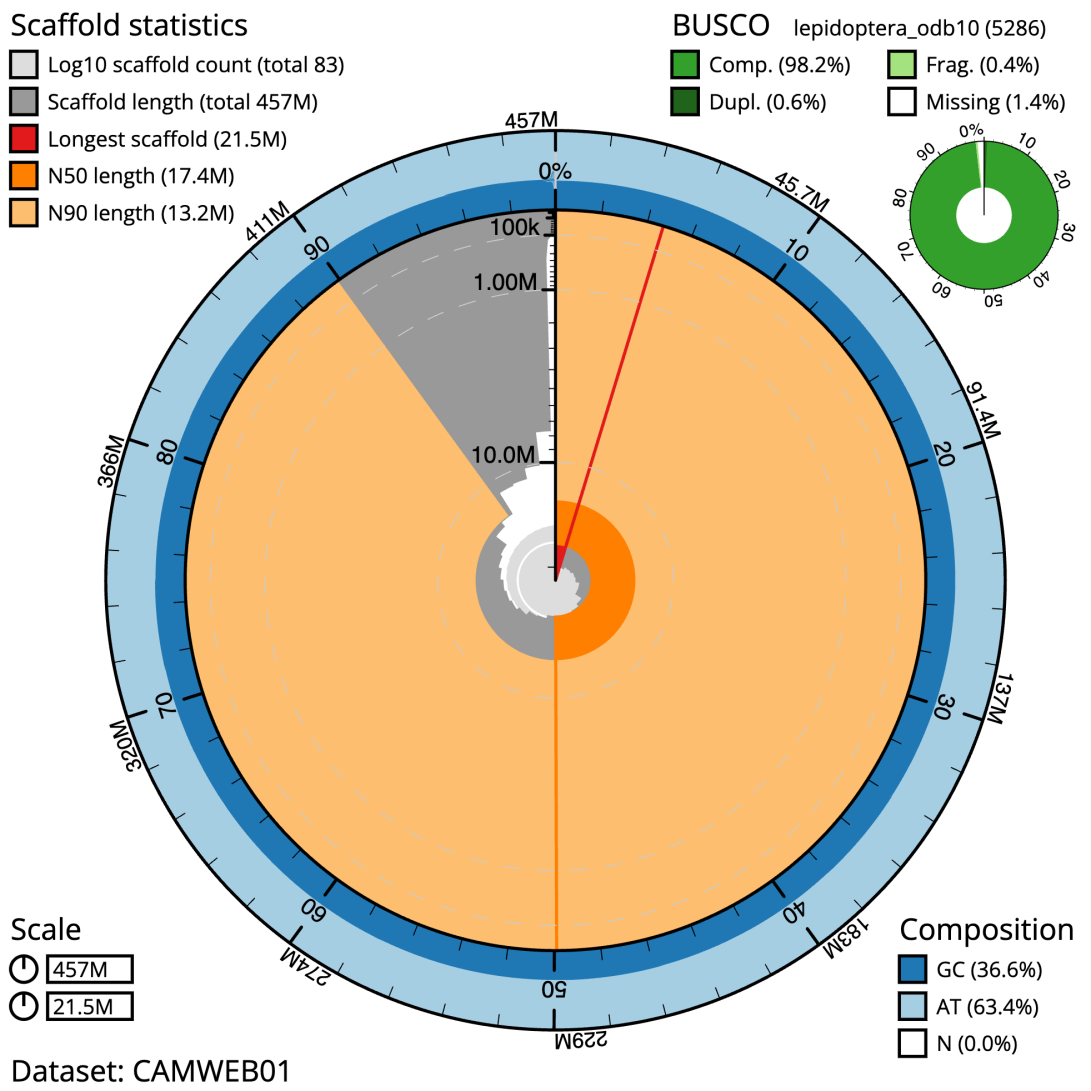
Genome assembly of *Bicyclus anynana*, ilBicAnyn1.1: metrics. The BlobToolKit Snailplot shows N50 metrics and BUSCO gene completeness. The main plot is divided into 1,000 size-ordered bins around the circumference with each bin representing 0.1% of the 457,175,899 bp assembly. The distribution of scaffold lengths is shown in dark grey with the plot radius scaled to the longest scaffold present in the assembly (21,498,244 bp, shown in red). Orange and pale-orange arcs show the N50 and N90 scaffold lengths (17,439,807 and 13,224,086 bp), respectively. The pale grey spiral shows the cumulative scaffold count on a log scale with white scale lines showing successive orders of magnitude. The blue and pale-blue area around the outside of the plot shows the distribution of GC, AT and N percentages in the same bins as the inner plot. A summary of complete, fragmented, duplicated and missing BUSCO genes in the lepidoptera_odb10 set is shown in the top right. An interactive version of this figure is available at https://blobtoolkit.genomehubs.org/view/ilBicAnyn1.1/dataset/CAMWEB01/snail.

The final assembly has a total length of 457.2 Mb in 83 sequence scaffolds with a scaffold N50 of 17.4 Mb ( Table 1). Most (99.51%) of the assembly sequence was assigned to 28 chromosomal-level scaffolds, representing 27 autosomes and the Z sex chromosome. Chromosome-scale scaffolds confirmed by the Hi-C data are named in order of size ( Figure 2– Figure 5; Table 2). While not fully phased, the assembly deposited is of one haplotype. Contigs corresponding to the second haplotype have also been deposited. The mitochondrial genome was also assembled and can be found as a contig within the multifasta file of the genome submission.

**Table 1.** Genome data for *Bicyclus anynana*, ilBicAnyn1.1.

Project accession data		
Assembly identifier	ilBicAnyn1.1	
Species	*Bicyclus anynana*	
Specimen	ilBicAnyn1	
NCBI taxonomy ID	110368	
BioProject	PRJEB54938	
BioSample ID	SAMEA9252597	
Isolate information	ilBicAnyn1, female (genome sequencing) ilBicAnyn2, male (Hi-C scaffolding) ilBicAnyn3, female (RNA sequencing)	
Assembly metrics *		*Benchmark*
Consensus quality (QV)	63	*≥ 50*
*k*-mer completeness	100%	*≥ 95%*
BUSCO **	C:98.2%[S:97.6%,D:0.6%], F:0.4%,M:1.4%,n:5,286	*C ≥ 95%*
Percentage of assembly mapped to chromosomes	99.51%	*≥ 95%*
Sex chromosomes	Z chromosome	*localised homologous pairs*
Organelles	Mitochondrial genome assembled	*complete single alleles*
Raw data accessions		
PacificBiosciences SEQUEL II	ERR10008895, ERR10008896, ERR10008897	
Hi-C Illumina	ERR9988140	
PolyA RNA-Seq Illumina	ERR10378024	
Genome assembly		
Assembly accession	GCA_947172395.1	
*Accession of alternate haplotype*	GCA_947172465.1	
Span (Mb)	457.2	
Number of contigs	770	
Contig N50 length (Mb)	1.2	
Number of scaffolds	83	
Scaffold N50 length (Mb)	17.4	
Longest scaffold (Mb)	21.5	

* Assembly metric benchmarks are adapted from column VGP-2020 of “Table 1: Proposed standards and metrics for defining genome assembly quality” from ( Rhie *et al.*, 2021). ** BUSCO scores based on the lepidoptera_odb10 BUSCO set using 5.3.2. C = complete [S = single copy, D = duplicated], F = fragmented, M = missing, n = number of orthologues in comparison. A full set of BUSCO scores is available at https://blobtoolkit.genomehubs.org/view/ilBicAnyn1.1/dataset/CAMWEB01/busco.

**Figure 3.**
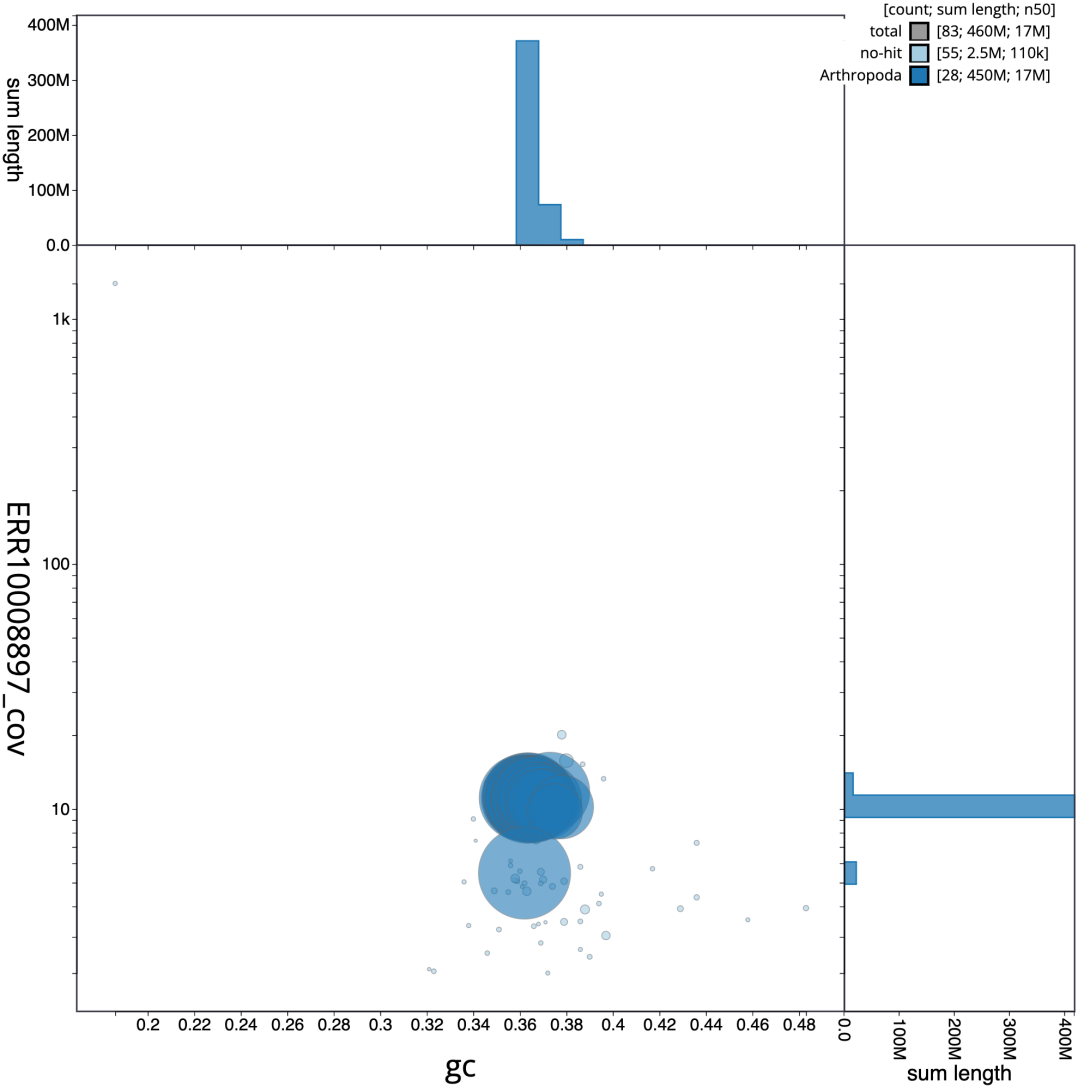
Genome assembly of *Bicyclus anynana*, ilBicAnyn1.1: BlobToolKit GC-coverage plot. Scaffolds are coloured by phylum. Circles are sized in proportion to scaffold length. Histograms show the distribution of scaffold length sum along each axis. An interactive version of this figure is available at https://blobtoolkit.genomehubs.org/view/ilBicAnyn1.1/dataset/CAMWEB01/blob.

**Figure 4.**
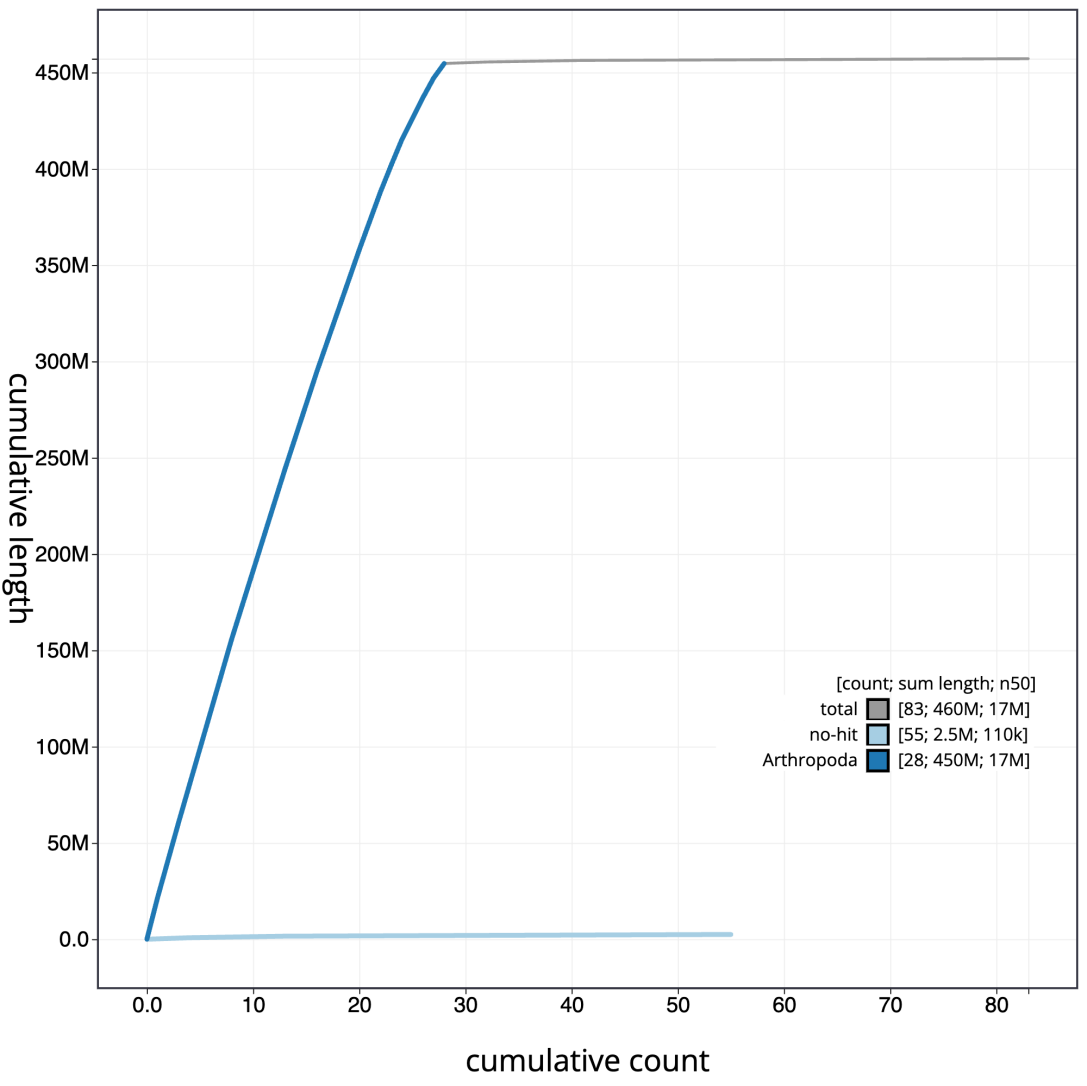
Genome assembly of *Bicyclus anynana*, ilBicAnyn1.1: BlobToolKit cumulative sequence plot. The grey line shows cumulative length for all scaffolds. Coloured lines show cumulative lengths of scaffolds assigned to each phylum using the buscogenes taxrule. An interactive version of this figure is available at https://blobtoolkit.genomehubs.org/view/ilBicAnyn1.1/dataset/CAMWEB01/cumulative.

**Figure 5.**
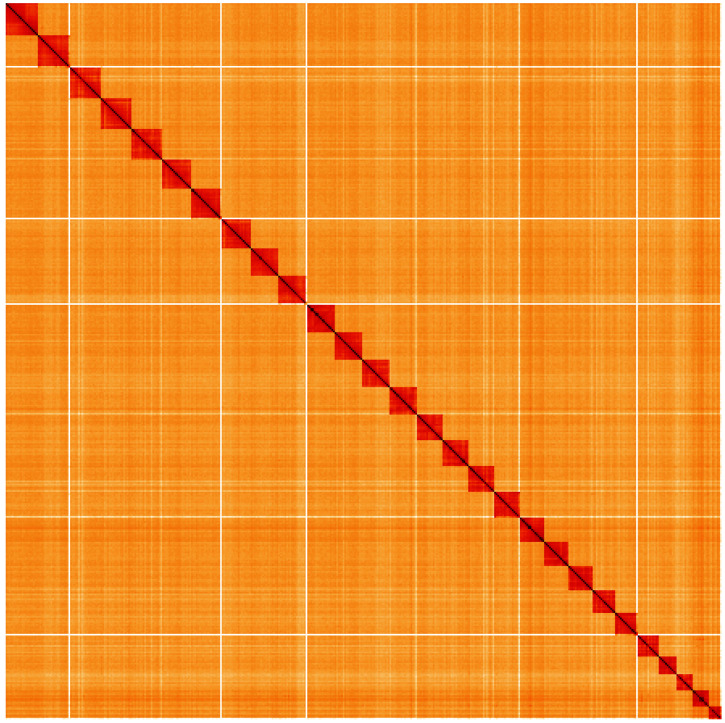
Genome assembly of *Bicyclus anynana*, ilBicAnyn1.1: Hi-C contact map of the ilBicAnyn1.1 assembly, visualised using HiGlass. Chromosomes are shown in order of size from left to right and top to bottom. An interactive version of this figure may be viewed at https://genome-note-higlass.tol.sanger.ac.uk/l/?d=TwYnm5nYQWuSaZneX5H9vQ.

**Table 2.** Chromosomal pseudomolecules in the genome assembly of *Bicyclus anynana*, ilBicAnyn1.

INSDC accession	Chromosome	Size (Mb)	GC%
OX359205.1	1	19.87	36.3
OX359206.1	2	19.55	36.5
OX359207.1	3	19.46	36.4
OX359208.1	4	19.23	36.4
OX359209.1	5	19.12	36.4
OX359210.1	6	18.7	36.2
OX359211.1	7	18.69	36.1
OX359212.1	8	17.88	36.6
OX359213.1	9	17.57	36.3
OX359214.1	10	17.51	36.2
OX359215.1	11	17.49	36.6
OX359216.1	12	17.44	36.3
OX359217.1	13	17.11	36.3
OX359218.1	14	16.98	36.5
OX359219.1	15	16.48	36.5
OX359220.1	16	16.15	36.5
OX359221.1	17	15.96	36.7
OX359222.1	18	15.78	37.3
OX359223.1	19	15.49	36.7
OX359224.1	20	15.16	36.6
OX359225.1	21	14.96	37
OX359226.1	22	13.7	36.7
OX359227.1	23	13.22	36.9
OX359228.1	24	11.08	37.1
OX359229.1	25	10.94	36.9
OX359230.1	26	10.07	37.8
OX359231.1	27	7.6	37.5
OX359204.1	Z	21.5	36.2
OX359232.1	MT	0.02	19.2
-	unplaced	2.47	37.7

The estimated Quality Value (QV) of the final assembly is 63 with *k*-mer completeness of 100%, and the assembly has a BUSCO v5.3.2 completeness of 98.2% (single = 97.6%, duplicated = 0.6%), using the lepidoptera_odb10 reference set ( *n* = 5,286).

Metadata for specimens, spectral estimates, sequencing runs, contaminants and pre-curation assembly statistics can be found at https://links.tol.sanger.ac.uk/species/110368.

## Methods

### Sample acquisition and nucleic acid extraction

Three *Bicyclus anynana* (ilBicAnyn1, ilBicAnyn2 and ilBicAnyn3) were collected from laboratory stock at the University of Liverpool on 15 August 2015. The specimens were killed and stored in a –80°C freezer.

The samples were prepared and DNA was extracted at the Tree of Life laboratory, Wellcome Sanger Institute (WSI). The ilBicAnyn1 sample was weighed and dissected on dry ice. Whole organism tissue was disrupted using a Nippi Powermasher fitted with a BioMasher pestle. High molecular weight (HMW) DNA was extracted using the Qiagen MagAttract HMW DNA extraction kit. HMW DNA was sheared into an average fragment size of 12–20 kb in a Megaruptor 3 system with speed setting 30. Sheared DNA was purified by solid-phase reversible immobilisation using AMPure PB beads with a 1.8X ratio of beads to sample to remove the shorter fragments and concentrate the DNA sample. The concentration of the sheared and purified DNA was assessed using a Nanodrop spectrophotometer and Qubit Fluorometer and Qubit dsDNA High Sensitivity Assay kit. Fragment size distribution was evaluated by running the sample on the FemtoPulse system.

RNA was extracted from whole organism tissue of ilBicAnyn3 in the Tree of Life Laboratory at the WSI using TRIzol, according to the manufacturer’s instructions. RNA was then eluted in 50 μl RNAse-free water and its concentration assessed using a Nanodrop spectrophotometer and Qubit Fluorometer using the Qubit RNA Broad-Range (BR) Assay kit. Analysis of the integrity of the RNA was done using Agilent RNA 6000 Pico Kit and Eukaryotic Total RNA assay.

### Sequencing

Pacific Biosciences HiFi circular consensus DNA sequencing libraries were constructed according to the manufacturers’ instructions. Poly(A) RNA-Seq libraries were constructed using the NEB Ultra II RNA Library Prep kit. DNA and RNA sequencing were performed by the Scientific Operations core at the WSI on Pacific Biosciences SEQUEL II (HiFi) and Illumina NovaSeq 6000 (RNA-Seq) instruments. Hi-C data were also generated from whole organism tissue of ilBicAnyn2 using the Arima2 kit and sequenced on the Illumina NovaSeq 6000 instrument.

### Genome assembly, curation and evaluation

Assembly was carried out with Hifiasm ( Cheng *et al.*, 2021) and haplotypic duplication was identified and removed with purge_dups ( Guan *et al.*, 2020). The assembly was then scaffolded with Hi-C data ( Rao *et al.*, 2014) using YaHS ( Zhou *et al.*, 2023). The assembly was checked for contamination as described previously ( Howe *et al.*, 2021). Manual curation was performed using HiGlass ( Kerpedjiev *et al.*, 2018) and Pretext ( Harry, 2022). The mitochondrial genome was assembled using MitoHiFi ( Uliano-Silva *et al.*, 2022), which runs MitoFinder ( Allio *et al.*, 2020) or MITOS ( Bernt *et al.*, 2013) and uses these annotations to select the final mitochondrial contig and to ensure the general quality of the sequence.

A Hi-C map for the final assembly was produced using bwa-mem2 ( Vasimuddin *et al.*, 2019) in the Cooler file format ( Abdennur & Mirny, 2020). To assess the assembly metrics, the *k*-mer completeness and QV consensus quality values were calculated in Merqury ( Rhie *et al.*, 2020). This work was done using Nextflow ( Di Tommaso *et al.*, 2017) DSL2 pipelines “sanger-tol/readmapping” ( Surana *et al.,* 2023a) and “sanger-tol/genomenote” ( Surana *et al.*, 2023b). The genome was analysed within the BlobToolKit environment ( Challis *et al.*, 2020) and BUSCO scores ( Manni *et al.*, 2021; Simão *et al.*, 2015) were calculated.

Table 3 contains a list of relevant software tool versions and sources.

**Table 3.** Software tools: versions and sources.

Software tool	Version	Source
BlobToolKit	4.0.7	https://github.com/blobtoolkit/blobtoolkit
BUSCO	5.3.2	https://gitlab.com/ezlab/busco
Hifiasm	0.16.1-r375	https://github.com/chhylp123/hifiasm
HiGlass	1.11.6	https://github.com/higlass/higlass
Merqury	MerquryFK	https://github.com/thegenemyers/MERQURY.FK
MitoHiFi	2	https://github.com/marcelauliano/MitoHiFi
PretextView	0.2	https://github.com/wtsi-hpag/PretextView
purge_dups	1.2.3	https://github.com/dfguan/purge_dups
sanger-tol/genomenote	v1.0	https://github.com/sanger-tol/genomenote
sanger-tol/readmapping	1.1.0	https://github.com/sanger-tol/readmapping/tree/1.1.0
YaHS	yahs-1.1.91eebc2	https://github.com/c-zhou/yahs

### Ethics and compliance issues

The materials that have contributed to this genome note have been supplied by a Darwin Tree of Life Partner. The submission of materials by a Darwin Tree of Life Partner is subject to the Darwin Tree of Life Project Sampling Code of Practice. By agreeing with and signing up to the Sampling Code of Practice, the Darwin Tree of Life Partner agrees they will meet the legal and ethical requirements and standards set out within this document in respect of all samples acquired for, and supplied to, the Darwin Tree of Life Project. Each transfer of samples is further undertaken according to a Research Collaboration Agreement or Material Transfer Agreement entered into by the Darwin Tree of Life Partner, Genome Research Limited (operating as the Wellcome Sanger Institute), and in some circumstances other Darwin Tree of Life collaborators.

## Data Availability

European Nucleotide Archive: *Bicyclus anynana* (squinting bush brown). Accession number PRJEB54938; https://identifiers.org/ena.embl/PRJEB54938 ( Wellcome Sanger Institute, 2022) The genome sequence is released openly for reuse. The *Bicyclus anynana* genome sequencing initiative is part of the Darwin Tree of Life (DToL) project. All raw sequence data and the assembly have been deposited in INSDC databases. The genome will be annotated using available RNA-Seq data and presented through the Ensembl pipeline at the European Bioinformatics Institute. Raw data and assembly accession identifiers are reported in Table 1.

## Author information

Members of the Wellcome Sanger Institute Tree of Life programme are listed here: https://doi.org/10.5281/zenodo.4783585.

Members of Wellcome Sanger Institute Scientific Operations: DNA Pipelines collective are listed here: https://doi.org/10.5281/zenodo.4790455.

Members of the Tree of Life Core Informatics collective are listed here: https://doi.org/10.5281/zenodo.5013541.

Members of the Darwin Tree of Life Consortium are listed here: https://doi.org/10.5281/zenodo.4783558.
