# Supplementary material for: The genome sequence of the Squinting Bush Brown, Bicyclus anynana (Butler, 1879)
Source: Wellcome Open Res. 2023 Jun 27;8:280. [Version 1] doi: 10.12688/wellcomeopenres.19432.1 (PMC10439359; doi:10.12688/wellcomeopenres.19432.1)
Supplement: Peer review history

# Peer review: Reviewer response for version 1

**DOI:** 10.21956/wellcomeopenres.21528.r64191

**Published:** 2023-08-18

**Authors:** Arnaud Martin

**Affiliations:** 1 Department of Biological Sciences, The George Washington University, Washington, District of Columbia, USA

Ilik Saccheri presents here a complete genome report for a high-quality genome assembly of Bicyclus anynana, a tropical butterfly that is also an important model system in the field of evo-devo.

 I applaud the author for generating this excellent resource, that is already empowering many important studies on the genetics of color patterning, seasonal plasticity, and adaptation.  I have no specific concern about this note, other than the following minor points in the **Data availability section.**

1. the report could mention the availability of the Genome as a RefSeq Genome at NCBI assembly 
2. the report currently mentions future annotation by EBI. However, it turns out it is already well annotated at the NCBI (see " https://www.ncbi.nlm.nih.gov/genome/annotation_euk/Bicyclus_anynana/GCF_947172395.1-RS_2022_12/")

Thus I believe the report should clearly mention something about the availability of the annotation generated "by the NCBI Eukaryotic Genome Annotation Pipeline".

 To avoid redundancy and save resources, I do not believe a second annotation attempt should be done by the EBI.

Are sufficient details of methods and materials provided to allow replication by others?

Yes

Is the rationale for creating the dataset(s) clearly described?

Yes

Are the datasets clearly presented in a useable and accessible format?

Yes

Are the protocols appropriate and is the work technically sound?

Yes

Reviewer Expertise:

Evolutionary Genetics

I confirm that I have read this submission and believe that I have an appropriate level of expertise to confirm that it is of an acceptable scientific standard.

# Peer review: Reviewer response for version 1

**DOI:** 10.21956/wellcomeopenres.21528.r64187

**Published:** 2023-08-18

**Authors:** Puteri Nur Syahzanani Jahari

**Affiliations:** 1 Department of Biosciences, Faculty of Science, Universiti Teknologi Malaysia, Skudai, Johor, Malaysia

The data note regarding the genome sequence of the Squinting Bush Brown butterfly ( *Bicyclus anynana*) is both clear and concise. Given that this species showcases seasonally plastic phenotypic characteristics, having access to its genome sequence holds significant potential for investigating the species' responses to different seasons. Advanced technologies have been used to complement the genome sequences with the chromosome information. Notably, the mitogenome of the species has also been successfully assembled. The assembly resulted in a high-quality genome (BUSCO > 98%) and highly mapped to the chromosome (> 99%). Thus, the reported protocols of sample extraction, sequencing, assembly and QC would be an ideal reference for future genomic studies.

Are sufficient details of methods and materials provided to allow replication by others?

Yes

Is the rationale for creating the dataset(s) clearly described?

Yes

Are the datasets clearly presented in a useable and accessible format?

Yes

Are the protocols appropriate and is the work technically sound?

Yes

Reviewer Expertise:

Animal genomics, evolutionary genetics

I confirm that I have read this submission and believe that I have an appropriate level of expertise to confirm that it is of an acceptable scientific standard.
